# Visualization of anatomical structures in the carpal region of the horse using cone beam computed tomography in comparison with conventional multidetector computed tomography

**DOI:** 10.3389/fvets.2024.1431777

**Published:** 2024-11-11

**Authors:** M. Hagenbach, J. Bierau, A. M. Cruz, C. Koch, G. Manso-Díaz, K. Büttner, C. Staszyk, M. Röcken

**Affiliations:** ^1^Equine Clinic (Surgery, Orthopedics), Justus-Liebig-University Giessen, Giessen, Germany; ^2^Department of Clinical Veterinary Medicine, Vetsuisse Faculty, Swiss Institute of Equine Medicine (ISME), University of Bern, Bern, Switzerland; ^3^Department of Animal Medicine and Surgery, Faculty of Veterinary Medicine, Universidad Complutense de Madrid, Madrid, Spain; ^4^Unit for Biomathematics and Data Processing, Justus-Liebig-University Giessen, Giessen, Germany; ^5^Institute of Veterinary-Anatomy, -Histology and -Embryology, Faculty of Veterinary Medicine, Justus-Liebig-University Giessen, Giessen, Germany

**Keywords:** horse, carpal joint, cone beam computed tomography, multidetector computed tomography, anatomy, diagnostic

## Abstract

**Introduction:**

In the diagnostics of orthopedic diseases in the horse, diagnostic imaging often plays a decisive role. Cone beam computed tomography (CBCT) imaging is used in both human and small animal medicine and becoming increasingly popular. To see whether CBCT imaging can be useful in the diagnosis of orthopedic diseases of the carpal region of the horse and to explore possible limitations we compared CBCT images with multidetector computed tomography (MDCT) images of the carpal region of equine cadaveric specimens.

**Materials and methods:**

Twenty-eight forelimbs from fifteen horses, slaughtered for reasons unrelated to this study, were examined. Native and contrast enhanced CBCT and MDCT scans were performed. Anatomical structures were blindly evaluated by three independent experienced observers using a visual scoring system previously reported and adapted to the equine carpal region. A descriptive evaluation was carried out as well as Spearman’s rank correlation and interobserver agreement was shown by percent agreement (PA).

**Results:**

Visualization of osseous structures was excellent in both MDCT and CBCT. Articular cartilage could only be assessed in contrast enhanced scans whereby MDCT showed a slightly better visualization than CBCT. Soft tissue structures were generally difficult to assess. An exception were the medial and lateral palmar intercarpal ligament, which could not be visualized in native but were well visualized in contrast enhanced scans in both MDCT and CBCT images.

**Discussion/conclusion:**

For the evaluation of osseous structures and some intraarticular ligaments after contrast enhancement, CBCT serves as a reliable diagnostic imaging modality for the equine carpal region. However, soft tissue structures and cartilage are imaged more reliably using MDCT.

## 1 Introduction

Diseases of the carpal region are a common source of lameness and loss of performance, especially in racehorses (1–4). In a cohort of 114 Standardbred racehorses in training, lameness was localized to the carpal region in 28% of the horses, overall, and in 56% of the horses with forelimb lameness, making carpal lameness most common in this study population (5). The most commonly described reasons for carpal lameness are developmental (e.g., incomplete ossification), degenerative, inflammatory, infectious, or traumatic (e.g., carpal fractures) insults (1, 6). The carpal joint represents a complex anatomic region with numerous osseous and non-calcified structures, i.e., cartilaginous elements, intra- and extraarticular ligaments as well as tendons and tendon sheaths (7). Different diagnostic imaging modalities are employed in the diagnostic workup of the carpal region in horses. In practice, radiographic projections of the extended as well as of the flexed carpus are taken, but diagnostic yield is limited due to superimposition of relevant anatomical structures. To avoid this, cross-sectional imaging modalities are gaining practical relevance in the diagnosis of carpal problems. In a previous study (8) of imaging of articular cartilage lesions, computed tomography (CT) arthrography showed the highest sensitivity (69,9%), followed by magnetic resonance (MR) arthrography (53,5%). The intraarticular injection of contrast medium in the antebrachiocarpal and middle carpal joints significantly improved the visibility of these lesions. The majority of comparative imaging studies in horses focuses on comparing magnetic resonance imaging (MRI), multidetector computed tomography (MDCT), and digital radiography (9). Taking a closer look at the different CT technologies currently in use for examinations of the equine carpal region, the conventional MDCT and cone beam computed tomography (CBCT) are to be considered. In MDCT imaging, a fan-shaped x-ray beam is rotated in a helical progression around the area of interest. For this the patient is moved at consistent speed in relation to and through the CT-gantry, producing image planes slice by slice. These slices are subsequently assembled into a 3D-reconstruction (10). In CBCT imaging, on the other hand, a fixed cone-shaped x-ray beam is projected onto a flat panel detector, while rotated around the region of interest within a gantry that remains in a fixed position in relation to the patient. The thereby acquired volumetric data is sampled with multiple projections of the complete field of view (FOV) from just a single rotation (11–13).

CBCT has its origins in various fields of human medicine such as angiography and intra-operative imaging procedures (14), radiotheraphy guidance (15), and mammography (16). Today, it finds widespread application in advanced dentistry and maxillofacial surgery (17) as well as image guided spine surgery (18). Additionally, CBCT imaging is used in diagnostic imaging of human extremities, allowing for rapid true to size visualization of osseous structures and to a lesser extend also of soft-tissue structures (19).

The progressive development of CBCT imaging in human medicine has brought forward numerous CBCT imaging units that are useful for application in veterinary medicine (9). Thus, for example, dental abnormalities in cats and dogs were examined with CBCT imaging (20, 21). Moreover, CBCT technology was described for the assessment of osseous maxillofacial structures and dentition in rabbits (22, 23).

Although the general diagnostic potential of standing CBCT for the equine carpal region has been mentioned (24), no systemic evaluation of the CBCT data quality exists. Thus, the aim of this study was to assess and evaluate clinically relevant structures of the equine carpal region in a comparative study using CBCT and MDCT scans.

## 2 Materials and methods

### 2.1 Cadaveric specimens

Twenty-eight forelimbs from fifteen horses, slaughtered for reasons unrelated to this study, were examined (Table 1). Before separating the limbs at mid-antebrachium level, a cable tie was fastened approximately 100 mm proximal of the antebrachiocarpal joint to avoid entrapment of air. After separation, the limbs were clipped and cleaned before radiographs were projected in four different planes (dorso-palmar, latero-medial, dorsolateral-palmaromedial oblique and dorsomedial-palmarolateral oblique) using a high-frequency generator (Siemens Optitop 150/40/80, 70 kV and 2.5 mAs) and a DR flat panel detector (Fujifilm, FDR D-EVO II C24). The specimens were then stored for a maximum of 24 h at 4°C before CBCT and MDCT scans were carried out.

**Table 1:** Patient data.

Horse number	Carpus number	Age (years)	Breed	Sex	Leg
1	1	12	Warmblood	Gelding	Right forelimb
1	2	12	Warmblood	Gelding	Left forelimb
2	3	14	Tinker horse	Mare	Left forelimb
3	4	16	Thoroughbred	Mare	Right forelimb
3	5	16	Thoroughbred	Mare	Left forelimb
4	6	19	Warmblood	Gelding	Right forelimb
4	7	19	Warmblood	Gelding	Left forelimb
5	8	19	Warmblood	Mare	Right forelimb
5	9	19	Warmblood	Mare	Left forelimb
6	10	20	Pony	Mare	Right forelimb
6	11	20	Pony	Mare	Left forelimb
7	12	23	Warmblood	Mare	Right forelimb
7	13	23	Warmblood	Mare	Left forelimb
8	14	24	Haflinger	Mare	Right forelimb
8	15	24	Haflinger	Mare	Left forelimb
9	16	25	Warmblood	Mare	Right forelimb
9	17	25	Warmblood	Mare	Left forelimb
10	18	25	Noriker	Gelding	Right forelimb
10	19	25	Noriker	Gelding	Left forelimb
11	20	26	Icelandic horse	Mare	Right forelimb
11	21	26	Icelandic horse	Mare	Left forelimb
12	22	26	Icelandic horse	Mare	Right forelimb
12	23	26	Icelandic horse	Mare	Left forelimb
13	24	27	Warmblood	Gelding	Right forelimb
13	25	27	Warmblood	Gelding	Left forelimb
14	26	28	Haflinger	Mare	Right forelimb
14	27	28	Haflinger	Mare	Left forelimb
15	28	29	Quarter horse	Gelding	Left forelimb

### 2.2 CBCT and MDCT scans

The FDA-approved CBCT scanner (O-arm^®^, Medtronic Inc.) for application in surgical environments was used in high definition (HD) mode with 120 kV, 120 mAs and a FOV of 200 mm. The FOV was large enough for all forelimbs examined. The MDCT scans were carried out with a helical 16-slice MDCT scanner (Somatom^®^ Definition AS Siemens, Erlangen, Germany). The device settings of 130 kV, 173 mAs, and 0,6 mm slices were applied for all image acquisitions with a FOV of 256 mm, running both soft tissue and bone algorithms.

First, a native CBCT scan and then a native MDCT scan were carried out with the limbs laying on the dorsal side. Subsequently, a 1:1 mixture of contrast medium (Xenetix^®^ 300, Guerbet, Sulzbach, Germany) and isotonic saline solution 0,9% (Braun Ecofl^®^, B. Braun Melsungen AG, Germany) was injected by dorsal approach in both the antebrachiocarpal (ACJ) and middle carpal joints (MCJ) with a 20 G cannula (Stercan^®^, B. Braun Melsungen AG, Germany) until the joints were ballooned (average volume 20 mL). Immediately afterwards the carpal joints were flexed and extended thirty times to get an even distribution of the injected solution. Subsequently, the limbs were scanned again with both MDCT and CBCT applying the same device settings as used for the native scans.

### 2.3 Image evaluation

**Figure 1:**
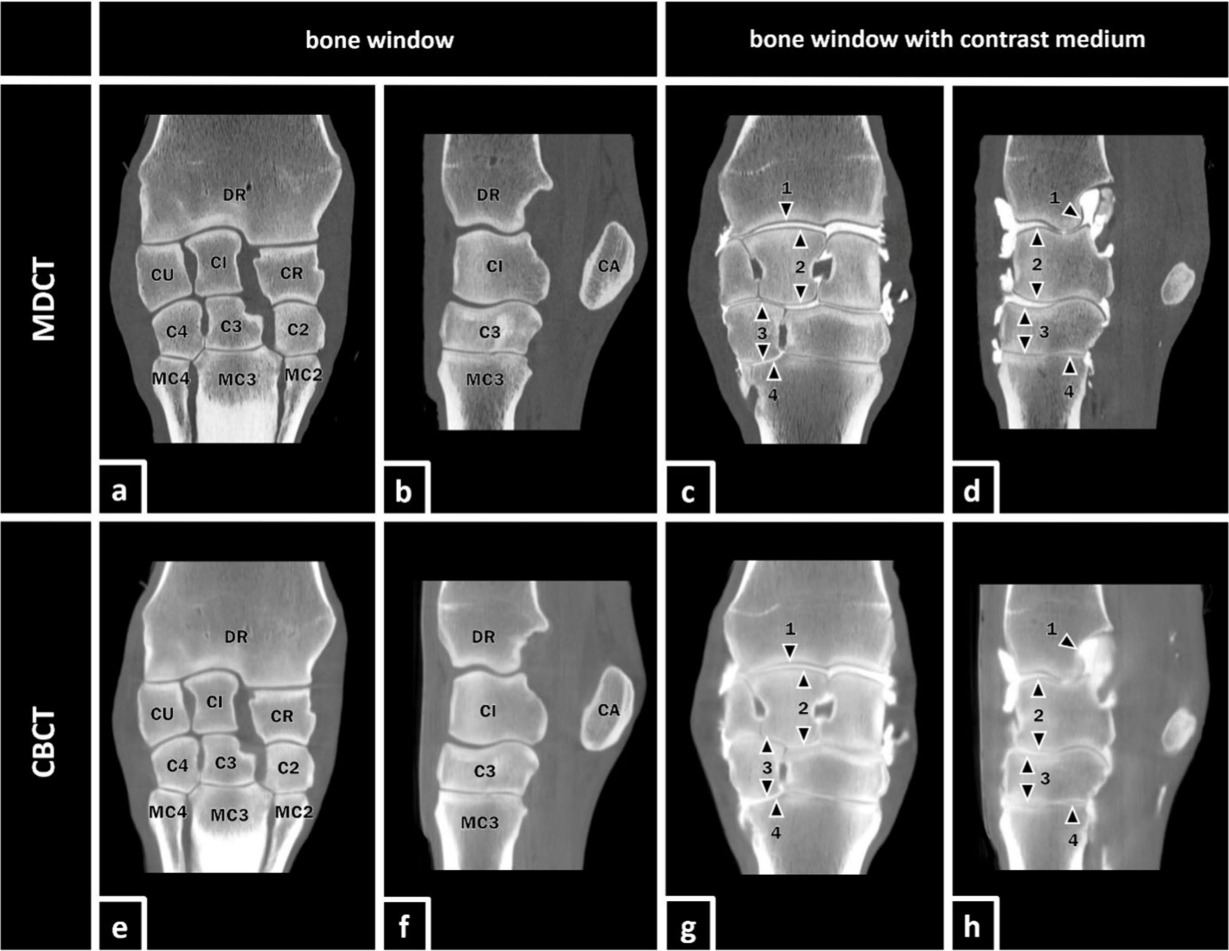
MDCT and CBCT images of the right forelimb of a 19-year-old gelding. The visibility of the listed osseous and articular structures was evaluated in MDCT and CBCT images with and without contrast enhancement. Osseous structures **(a,b,e,f)** (DR) distal radius, (CU) ulnar carpal bone, (CI) intermediate carpal bone, (CR) radial carpal bone, (CA) accessory carpal bone, (C4) fourth carpal bone, (C3) third carpal bone, (C2) second carpal bone, (MC4) fourth metacarpal bone, (MC3) third metacarpal bone, and (MC2) second metacarpal bone. Articular structures (**c,d,g,h**; marked with black arrows): (1) cartilage of DR, (2) cartilage of the antebrachial (proximal) row, (3) cartilage of the metacarpal (distal) row, and (4) cartilage of proximal MC3.

A DICOM viewing software (DICOM Horos^®^ viewer) was used in a quiet and darkened examination room with the help of a MacBook Pro^®^, 2,3 GHz Dual-Core Intel Core i5, MacOs Ventura 13.4 to display the MDCT and CBCT scans while a multiplanar reconstruction tool was utilized for the different slice planes. Numbers were randomly given for each forelimb and the observers could allocate the two different modalities to the respective limb. All observers [two board-certified equine surgeons (CK, AC) and one board-certified large animal radiologist (GMD)] were experienced in interpreting MDCT and CBCT images. The observers were instructed to evaluate the visibility of the following clinically relevant structures using a scoring system adapted from Bierau et al. (25):

Osseous structures (Figure 1):

- Distal aspect of the radius (DR)
- Proximal (antebrachial) row of the carpal bones (incl. Accessory carpal bone) (AR)
- Distal (metacarpal) row of the carpal bones (MR)
- Proximal aspects of the metacarpal bones (PMC)

Articular structures (Figure 1):

- Cartilage of DR
- Cartilage of AR
- Cartilage of MR
- Cartilage of PMC

**Figure 2:**
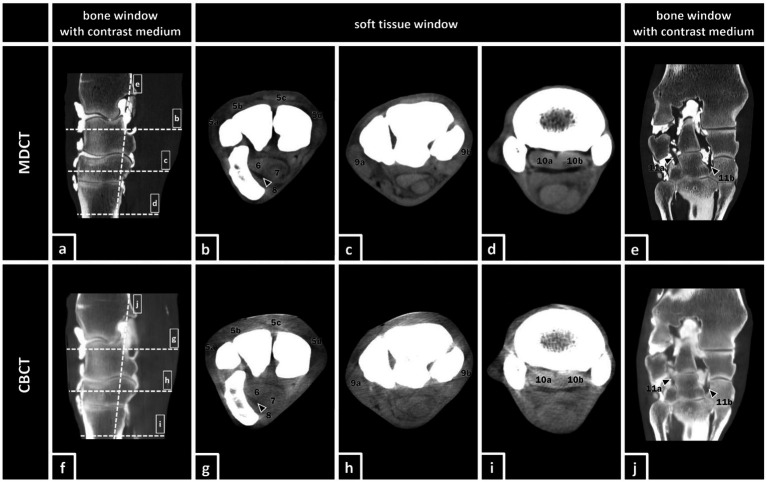
MDCT and CBCT images of the right forelimb of a 19-year-old gelding. The visibility of the listed soft tissue structures was evaluated in MDCT and CBCT images with and without contrast enhancement. Soft tissue structures: (5a) lateral digital extensor tendon, (5b) dorsal (common) digital extensor tendon, (5c) extensor carpi radialis tendon, (5d) abductor pollicis longus tendon, (6) deep digital flexor tendon (DDFT), (7) superficial digital flexor tendon (SDFT), (8) carpal flexor tendon sheath (CFTS), (9a) lateral collateral ligament of the carpus, (9b) medial collateral ligament of the carpus, (10a+b) origin of the suspensory ligament (OSL), (10a) lateral lobe, (10b) medial lobe, (11a) lateral palmar intercarpal ligament (LPIL), and (11b) medial palmar intercarpal ligament (MPIL).

Soft tissue structures (Figure 2):

- Digital extensor tendons (DET)
- Deep digital flexor tendon (DDFT)
- Superficial digital flexor tendon (SDFT)
- Carpal flexor tendon sheath (CFTS)
- Collateral ligaments of the carpus (CL)
- Medial palmar intercarpal ligament (MPIL)
- Lateral palmar intercarpal ligament (LPIL)
- Origin of the suspensory ligament (OSL)

**Figure 3:**
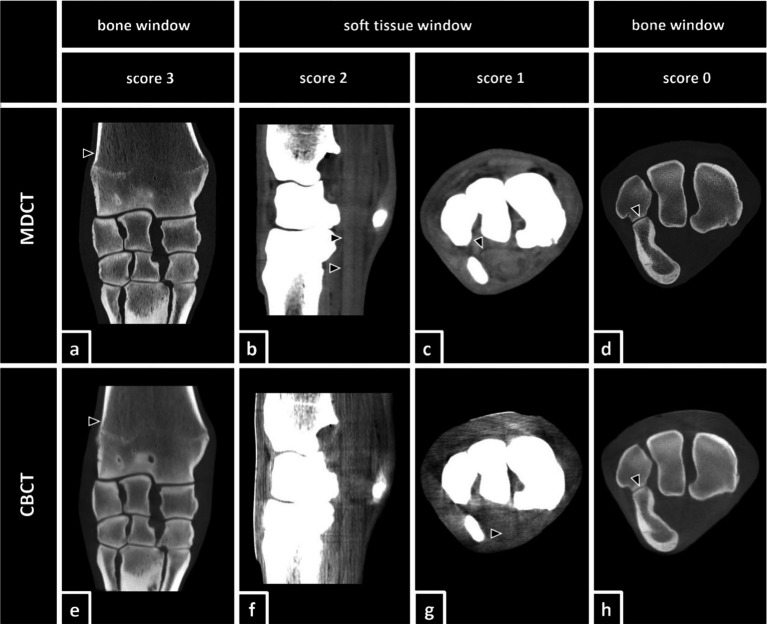
Definition of the utilized scoring system according to Vallance et al. (26) and Bierau et al. (25) including examples of differently scored anatomical structures. Score 3: **(a,e)** The distal aspect of the radius (black arrows) was clearly visualized and delineated by its location, shape, attenuation, size, and margin in both, MDCT and CBCT. Score 2: **(b)** In MDCT, the deep digital flexor tendon (black arrows) was clearly identified by location, shape, and attenuation, but the margins were not clearly delineated. In the accompanying CBCT image **(f)**, none of the structures was scored 2. Score 1: **(c)** In MDCT, the carpal flexor tendon sheath (black arrow) was poorly visualized, but detectable, and was identified by its location and attenuation but not by margins, shape, or size. In the accompanying CBCT image **(g)**, the superficial digital flexor tendon (black arrow) was scored 1. Score 0: **(d,h)** Although the lining of the cortical bone (black arrows) of the ulnar carpal bone and the accessory carpal bone was clearly visible, the belonging cartilage remained invisible.

For the evaluation of the images, a modified visual scoring system according to Vallance et al. (26) and Bierau et al. (25) was used. The scoring system consists of visual assessment scores from zero to three using subjective criteria for visibility for each structure. A score value of zero indicates that the evaluated structure was not visible. If a structure was poorly visualized, only identified by its location and signal intensity but not by margins, shape, or size, the observers scored the structure with a score value of one. A score value of two indicated that the structure could be clearly identified by its location, shape, and signal intensity but the margins were not clearly delineated. The highest score value of three represented a structure that was well visualized and clearly delineated by its location, shape, signal intensity, size, and margins (Figure 3).

### 2.4 Statistics

For the statistical analysis a statistical software (SAS^®^ 9.4) was used. A descriptive evaluation and comparison of the different recordings from the two devices before and after injection of contrast medium (MDCT native, CBCT native, MDCT contrast, and CBCT contrast) was performed belonging the visualization of the aforementioned anatomical structures (Table 2). The agreement of CBCT with MDCT was determined with the help of Spearman’s rank correlation coefficient (r_s_) and percent agreement (PA) represents the interobserver agreement. A correlation coefficient of $r_{s}$ > 0.4 represents acceptable agreement and $r_{s}$ > 0.7 represents good agreement. For the parameter percent agreement also a value close to 1 defines a good agreement between the different observers.

**Table 2:** Visualization score and technique comparison of cone beam computed tomography (CBCT) and conventional multidetector computed tomography (MDCT).

		Joint				Tendons/ Tendon sheaths				Ligaments			
Technique	Statistics	Cartilage DR	Cartilage AR	Cartilage MR	Cartilage PMC	DET	DDFT	SDFT	CFTS	CL	MPIL	LPIL	OSL
MDCT native, *n* = 84	Median (range)	0.00 (0–0)	0.00 (0–0)	0.00 (0–0)	0.00 (0–0)	2.00 (1–2)	2.00 (1–2)	2.00 (1–2)	1.00 (0–2)	1.00 (0–2)	0.00 (0–1)	0.00 (0–1)	2.00 (1–3)
	Mean	0.00	0.00	0.00	0.00	1.74	1.72	1.66	0.57	0.68	0.33	0.33	1.80
	Percent agreement	1.00	1.00	1.00	1.00	0.48	0.45	0.45	0.31	0.32	0.33	0.33	0.55
MDCT contrast, *n* = 84	Median (range)	3.00 (3–3)	3.00 (2–3)	2.00 (1–3)	1.00 (0–3)	1.00 (0–2)	2.00 (1–2)	2.00 (1–2)	1.00 (0–2)	0.00 (0–1)	3.00 (2–3)	3.00 (2–3)	2.00 (1–3)
	Mean	3.00	2.98	2.06	1.16	1.19	1.93	1.91	0.78	0.33	2.67	2.67	1.99
	Percent agreement	1.00	0.98	0.46	0.24	0.14	0.86	0.81	0.35	0.33	0.33	0.33	0.89
CBCT native, *n* = 84	Median (range)	0.00 (0–0)	0.00 (0–0)	0.00 (0–0)	0.00 (0–0)	0.00 (0–1)	1.00 (0–2)	1.00 (0–1)	0.00 (0–1)	0.00 (0–0)	0.00 (0–0)	0.00 (0–0)	1.00 (0–2)
	Mean	0.00	0.00	0.00	0.00	0.46	0.72	0.69	0.06	0.00	0.00	0.00	0.97
	Percent agreement	1.00	1.00	1.00	1.00	0.36	0.49	0.50	0.90	1.00	1.00	1.00	0.70
CBCT contrast, *n* = 84	Median (range)	2.00 (1–3)	2.00 (1–3)	1.00 (0–3)	1.00 (0–2)	0.00 (0–1)	0.00 (0–1)	0.00 (0–1)	0.00 (0–1)	0.00 (0–1)	3.00 (0–3)	3.00 (0–3)	1.00 (0–3)
	Mean	2.36	2.19	1.40	0.81	0.17	0.44	0.44	0.07	0.01	2.50	2.50	1.18
	Percent agreement	0.30	0.29	0.26	0.42	0.67	0.33	0.33	0.88	0.98	0.32	0.32	0.70
Comparisons:													
MDCT native vs. CBCT native	Spearman’s rank correlation	r_s_ =*	r_s_ =*	r_s_ =*	r_s_ =*	r_s_ = 0.23	r_s_ = 0.58	r_s_ = 0.57	r_s_ = 0.23	r_s_ =*	r_s_ =*	r_s_ =*	r_s_ = −0.02
	*p*-value	*p* =*	*p* =*	*p* =*	*p* =*	*p* = 0.04	*p* ≤ 0.01	p ≤ 0.01	*p* = 0.03	*p* =*	*p* =*	*p* =*	*p* = 0.89
MDCT contrast vs. CBCT contrast	Spearman’s rank correlation	r_s_ =*	r_s_ = −0.12	r_s_ = 0.54	r_s_ = 0.47	r_s_ = 0.15	r_s_ = −0.25	r_s_ = −0.36	r_s_ = 0.32	r_s_ = 0.16	r_s_ = 0.94	r_s_ = 0.94	r_s_ = 0.24
	*p*-value	*p* =*	*p* = 0.29	*p* ≤ 0.01	*p* ≤ 0.01	*p* = 0.18	*p* = 0.02	*p* ≤ 0.01	*p* ≤ 0.01	*p* = 0.16	*p* ≤ 0.01	*p* ≤ 0.01	*p* = 0.03

*The correlation coefficient could not be calculated, because there was not enough variance in the present data (see percent agreement). DR, distal aspect of the radius; AR, antebrachial (proximal) row of the carpal bones (incl. Accessory carpal bone); MR, metacarpal (distal) row of the carpal bones; PMC, proximal aspects of the metacarpal bones (MCII, III, IV); DET, digital extensor tendons; DDFT, deep digital flexor tendon; SDFT, superficial digital flexor tendon; CFTS, carpal flexor tendon sheath; CL, collateral ligaments; MPIL, medial palmar intercarpal ligament; LPIL, lateral palmar intercarpal ligament; OSL, origin of the suspensory ligament.

## 3 Results

### 3.1 Comparison of native MDCT and CBCT images

All osseous structures examined in this study (DR, AR, MR, PMC; in total *n* = 112) were well visualized in native CBCT as well as in native MDCT (MDCT and CBCT mean score 3.0, PA = 1). The cartilage of these structures was not be seen in any of the native scans (MDCT and CBCT mean score 0.0, PA = 1). In general, soft tissue structures were better visualized in MDCT than in CBCT scans. In detail, tendons/tendon sheaths (MDCT mean score 1.42, PA = 0.42 vs. CBCT mean score 0.49, PA = 0.56) were better visualized than ligaments (MDCT mean score 0.78, PA = 0.38 with r_s_ from 0.80 to 0.99 vs. CBCT mean score 0.24, PA = 0.93 with r_s_ from 0.84 to 0.93). Intracarpal ligaments (MPIL and LPIL) were not well visualized, neither in native CBCT (score 0.0, PA = 1) nor in native MDCT scans (mean score 0.33, PA = 0.33).

### 3.2 Comparison of native MDCT with contrast enhanced MDCT and native CBCT with contrast enhanced CBCT

The intraarticular injection of contrast medium did not change the visibility of osseous structures neither in MDCT (mean score 3.0, PA = 1) nor in CBCT (mean score 3.0, PA = 0.99). A significant improvement of the visualization of cartilage was shown after contrast enhancement in both MDCT (mean score 2.30, PA = 0.67 with r_s_ from 0.73 to 0.83) and CBCT (mean score = 1.69, PA = 0.32 with r_s_ from 0,45 to 0.61), whereby the visualization was better in MDCT than in CBCT. When focusing on the articular structures, the cartilage of DR was rated the highest (MDCT mean score 3.0, PA = 1 vs. CBCT mean score 2.38, PA = 0.30) while the cartilage of the PMC showed the lowest score (MDCT mean score 1.2, PA = 0.24 vs. CBCT mean score 0.86, PA = 0.42). Tendons and tendon sheaths had low scores in both MDCT and CBCT, where MDCT showed better visibility (mean score 1.45, PA = 0.54) than CBCT (mean score 0.28, PA = 0.55). The MPIL and LPIL, as intraarticular ligaments, were significantly better visualized after contrast enhancement (r_s_ = 0.94) in both MDCT (mean score 2.67, PA = 0.33) and CBCT (mean score 2.5, PA = 0.32), while CL could almost not be visualized (MDCT mean score 0.33, PA = 0.33 vs. CBCT mean score 0.01, PA = 0.98). The OSL was better visualized in MDCT (mean score 1.99, PA = 0.89) than in CBCT (mean score 1.18, PA = 0.70) but this did not improve significantly with contrast enhancement.

## 4 Discussion

In the present study, reconstructed CBCT and MDCT images of clinically relevant anatomical structures of the equine carpal region were acquired in fresh cadaveric specimens and compared with the help of a scoring system. While osseous structures were well visualized in both modalities before and after contrast enhancement, cartilage could only be seen after the injection of contrast medium. The visibility of cartilage after contrast enhancement was better in MDCT than in CBCT. For soft tissue structures such as ligaments, tendons and tendon sheaths, the MDCT images showed superior quality in general.

### 4.1 Conditions of CBCT

In this study, CBCT showed a similar capacity in the visualization of osseous structures compared to images produced by MDCT. After contrast enhancement, indirect visualization of cartilage improved for both modalities, but more so in MDCT. When focusing on the periarticular soft tissue structures, neither of the two modalities performed well, regardless of the use of contrast enhancement. However, following contrast enhancement, the MPIL and LPIL became well visualized in both modalities. This is explained by their intraarticular position and the corresponding possibility of an indirect representation when visualizing the synovial space, similar to the principle of indirect articular cartilage visualization by adding contrast medium to the synovia.

One of the disadvantages of CBCT imaging is the cone beam effect, which occurs because of the divergence of the cone-shaped x-ray beam. That’s why more information is recorded for centered structures than for peripheral objects and leads to more peripheral noise and other artefacts such as more scatter radiation, reduced contrast resolution and with that poorer soft tissue visualization (13, 27, 28). Motion artefacts are often described as a major factor for disruption and repetitions of examinations. Unlike in MDCT imaging, where motion artefacts are restricted to the segment that is being scanned at the time of patient movement, in CBCT all images reconstructed from the acquired volume will show motion artefacts, because the x-ray beam only rotates once around the subject of interest. Obviously, because this study was performed on cadaveric specimens, this limiting effect could not be assessed. However, based on clinical experience, motion artefacts are less frequently a limiting factor when scanning extremities compared to heads in standing CBCT imaging of horses and other equids (13, 24). Furthermore, post-processing motion correction software is under development (27). For the used CBCT scanner in this study (O-arm^®^ (Medtronic), O-arm caliber 699 mm), the FOV is limited for 397 × 160 mm with a resolution of 512 × 512 × 192 voxel. For some regions of interest, such as the equine head or the stifle joint, it can necessitate more than one scan to complete the whole examination (27). The scans of the carpal region in this study were even performed with the smaller cylindrical volume of 212 × 160 mm and we were able to confirm that this was sufficient for all examinations carried out.

### 4.2 CBCT vs. MDCT

The overall portrayal of the anatomic structures appears to be of higher quality when produced with MDCT scanners than when produced with CBCT scanners. However, particularly in regards to the visualization of osseous tissues, there appeared to be only minor differences between CBCT and MDCT imaging of the carpal joint of horses. Further investigations with known or provoked pathologies would have to be conducted to determine the effect on diagnostic yield and reliability when comparing the two modalities.

For computed tomographic arthrography of articular structures, MDCT showed better visualization than CBCT and the different soft tissue structures, except MPIL and LPIL, were difficult to assess with and without contrast enhancement and with both MDCT and CBCT (Table 2). Because two-dimensional imaging reaches its limits in such complex anatomical areas as the equine carpal joints, three-dimensional diagnostic imaging such as MRI, MDCT, and CBCT are the modalities of choice for comprehensive diagnostics. For the portrayal of cartilage lesions in equine carpal joints, computed tomographic arthrography showed a higher sensitivity than contrast-enhanced MRI scans (8), while the current study suggests that CBCT could not really join this category. Although CBCT deals with different disadvantages as mentioned before, the modality also has its merits that should be considered. Particular in regards to its practical advantages, CBCT imaging represents a valuable adjunctive and alternative modality to MDCT and other three-dimensional imaging modalities in horses. CBCT scans are mostly well tolerated even in standing sedated horses, probably because of low noise and the fast acquisition time, because neither the gantry nor the patient is moving during the examination (13). Furthermore, it can be applied both as a preoperative planning tool and due to its ability to be mobile as an intraoperative three-dimensional imaging modality in horses and lower radiation dose and acquisition costs compared to MDCT scanners are described as well as a slightly higher spatial resolution (27, 29). When it comes to the decision what kind of imaging modality should be used for different clinical cases, the diagnostic value of the chosen modality is of highest importance. However, the practicability, technical features, and purchase conditions should not be neglected either.

### 4.3 Visibility of MPIL and LPIL

**Figure 4:**
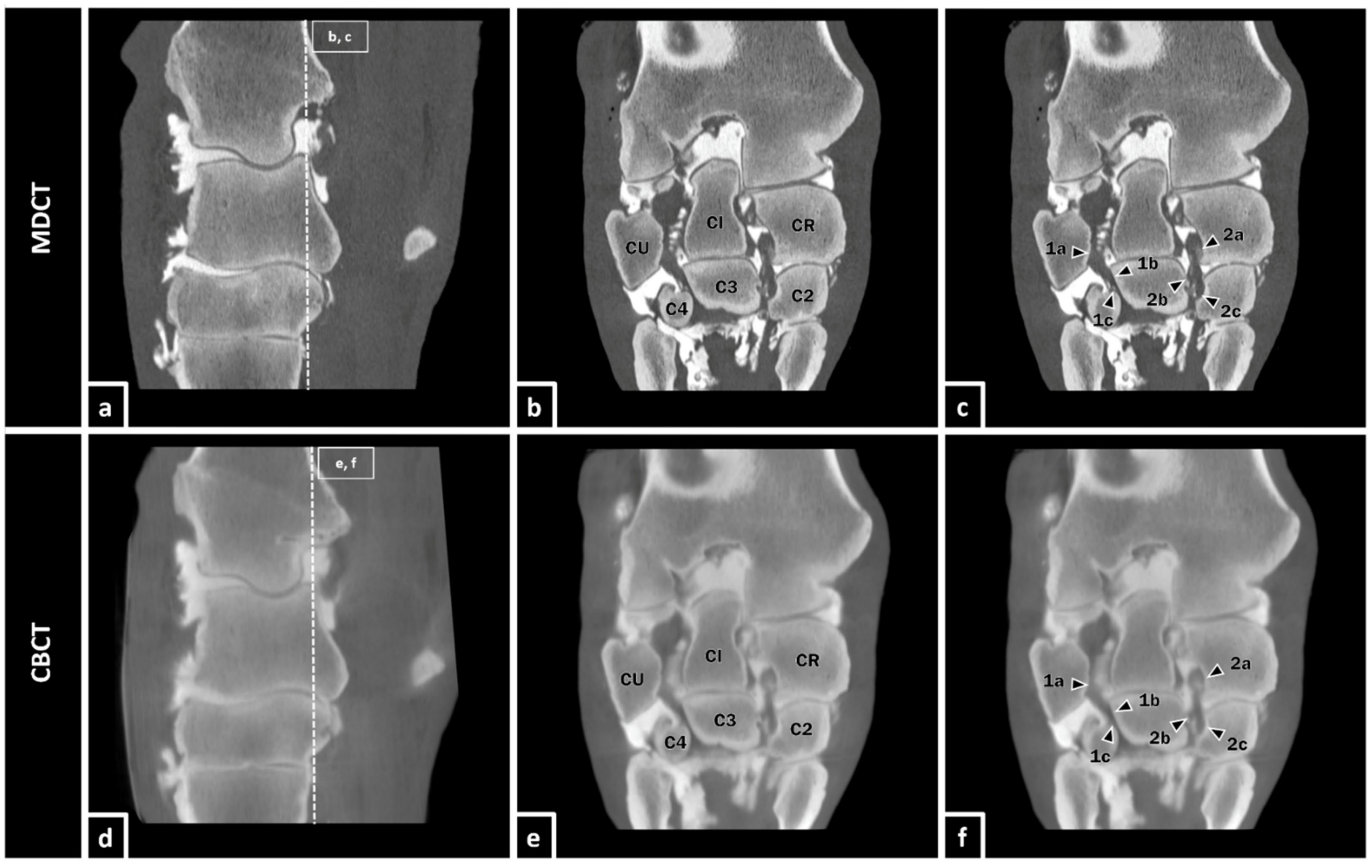
Equine carpal region in sagittal section **(a,d)**. Depiction of the MPIL and LPIL in coronal section as representants of the equine intracarpal ligaments in bone window after contrast enhancement in MDCT **(b,c)** and CBCT **(e,f)** of a 25-year-old gelding. The LPIL (1a-c) reaches from the distal part of the palmaromedial surface of the ulnar carpal bone (CU) to the proximal palmarolateral surface of the third carpal bone (C3) and also, with a few fibers, to the palmaromedial surface of the fourth carpal bone (C4). The MPIL (2a-c) ranges from the distolateral surface of the radial carpal bone (CR) to the proximal palmaromedial surface of the third carpal bone (C3) and the proximal palmarolateral aspect of the second carpal bone (C2) (37).

Because the MPIL and LPIL are reported as significant sources of carpal lameness and instability in horses (30–33), they were also part of this investigation. In a study of 1992, the number of horses with MPIL injuries was suspected to be much higher than previously thought (34) but one of the biggest challenges is the significant imaging representation. Although radiography and ultrasonography can be used to assess the MPIL and LPIL, these modalities are limited. Likewise, arthroscopy as a minimally-invasive diagnostic modality has its limitations in assessing the intercarpal ligaments because of the restricted window of visualization (30, 35). In contrast, MRI and CT arthrography are valuable diagnostic imaging modalities to assess the intercarpal ligaments (30, 36). The portrayal of MPIL and LPIL after contrast enhancement in CT corresponds well with that in MRI. However, MRI requires more scan time than CT imaging, making CT arthrography a valid alternative (30). This was confirmed in the present study, where the intercarpal ligaments became reliably visible after contrast enhancement in both MDCT and CBCT imaging (Figure 4). A possible reason for the relatively low interobserver agreement (PA = 0.33) for these two intraarticular ligaments could be that one observer scored these two ligaments with an MDCT mean score of 2.00 and CBCT mean score of 1.61 while the other two observers scored them with an MDCT mean score of 3.00 and CBCT mean score of 2.95. Nevertheless, even in MDCT as well as in CBCT, they showed a significant improvement of visualization after contrast enhancement (r_s_ = 0.94) overall. To our knowledge, there are no previous studies about the portrayal of MPIL and LPIL in CBCT imaging.

### 4.4 Communication between carpometacarpal joint and OSL

**Figure 5:**
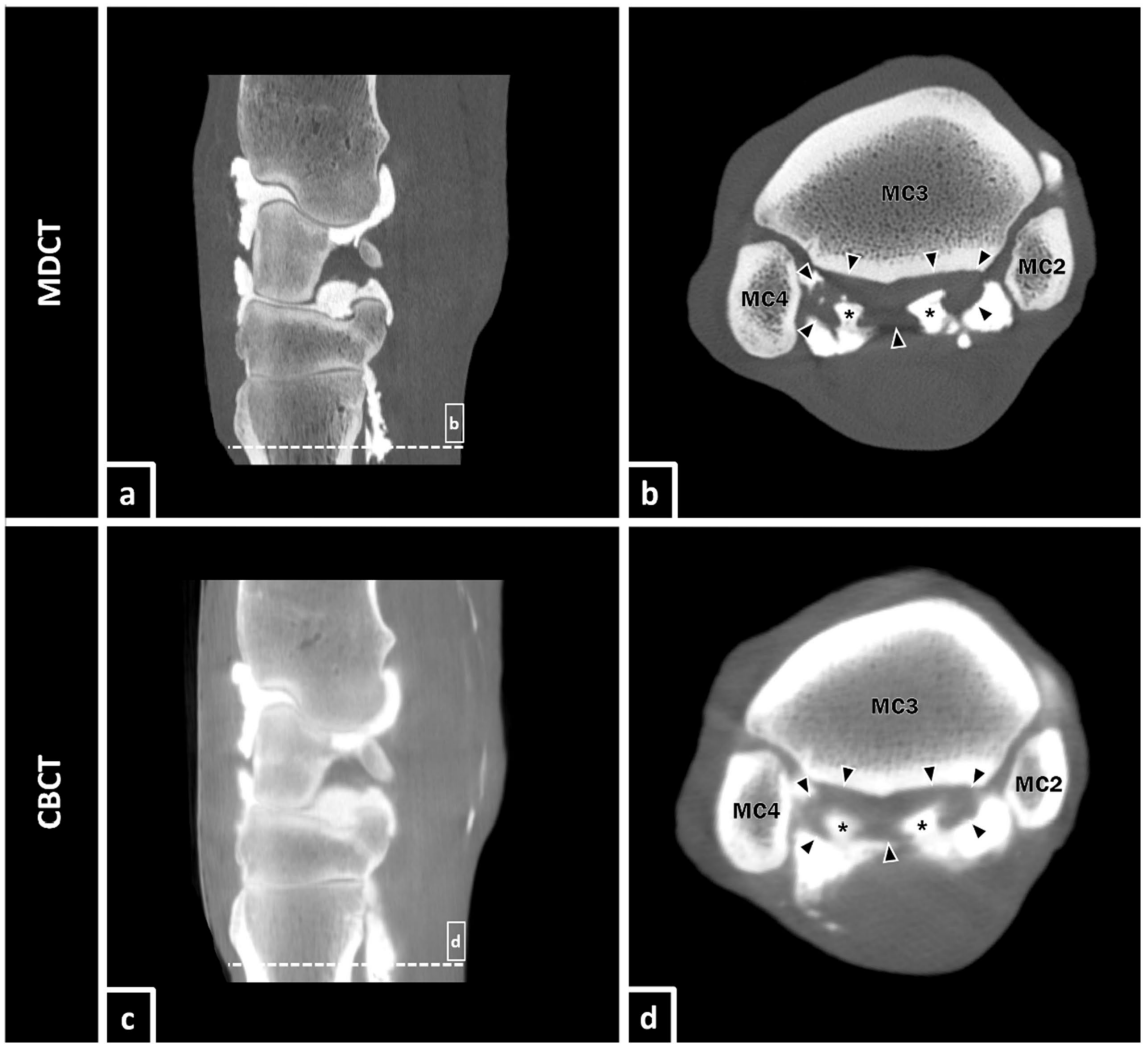
Equine carpal region in sagittal section **(a,c)**. Presence of positive contrast medium (asterisks) in the lateral and medial lobe of the proximal suspensory ligament (borders of the OSL are defined by black arrows) in transversal section **(b,d)** after injection of a mixture of contrast medium in the ACJ and MCJ. The lateral and medial palmarodistal outpouchings of the CMCJ are apparently filled with contrast medium too and it appears to exist a connection to the lobes of the OSL.

In this study, an enrichment of contrast medium in the OSL was observed in several forelimbs after intraarticular injection in the ACJ and MCJ (Figure 5). Even if the MDCT image and the CBCT image in this figure are at the same level, the contrast medium is already more widely distributed in CBCT than in MDCT. CBCT scans were performed immediately after MDCT scans, which may have resulted in a slightly different distribution of contrast medium. In previous studies, a connection of the OSL with the CMCJ could already be shown (30, 38). Communication between areas of the carpal joint and the OSL were also registered in this study, whereupon the clinical relevance needs to be examined in more detail in further studies. The accumulation of contrast medium in the OSL could be seen in both, MDCT and CBCT. It must be noted that contrast solutions used for diagnostic anesthesia or for intraarticular medication may have different properties and therefore behave differently in life animals with weight bearing extremities and compared with the mixture of contrast agents used in this study on cadaveric specimens.

### 4.5 Visibility of cartilage after contrast enhancement

Cartilage was only visualized indirectly after injection of contrast enhancement. Overall, the visibility of the cartilage in contrast-enhanced scans was slightly better in MDCT than in CBCT scans, but decreased from proximal to distal in both modalities. The joint capsule of the ACJ therefore has a wide dorsal recessus and has the greatest range of motion and thus cartilaginous surface area. Further distally is the MCJ, which contributes less to the range of motion of the carpus and is less voluminous. The articulating osseous structures of the CMCJ, the bones of the distal/metacarpal row of the carpal joint, and the metacarpal bones, offer flatter joint surfaces. These are tight joints with a narrow joint capsule and therefore almost no room for joint movement (39). These morphological particularities together with the fact of thinner cartilage in the distal joint compartments (40) explain the poorer visibility of articular cartilage in distal carpal aspects. However, in terms of clinical relevance, this observation can probably be neglected, since the pathological changes in the articular structures tend to occur in the more proximal areas with more mobility and movement-induced stress (5–7).

### 4.6 Impact of evaluation

In this study, an adapted scoring system was used to compare CBCT and MDCT images with each other and among the different observers. It is about a subjective assessment as there is no objective data for comparison. In evaluation of MDCT images, Hounsfield units (HU) are used to identify structural changes and even only slight differences. A comparable parameter in CBCT is shown by gray scale (voxel value), but even if there is a strong correlation between HU in MDCT and gray levels in CBCT, it is not possible to directly convert HU in gray levels of CBCT due to the high influence of artefacts in CBCT (41). In addition, different reconstruction algorithms make a direct objective comparison impossible. This is due to technical differences between the two modalities.

For the anatomical structures in which a percent agreement of one and therefore no variance in the scores could be achieved, no r_s_ could be calculated, which does not mean that the r_s_ is equal to zero or one, but that the agreement is 100% and the r_s_ could not be calculated due to a lack of variance of the scores [all zero (for example category joint native CBCT as well as MDCT) or all three (for example category osseous structures native and contrast, CBCT as well as MDCT)]. This has to be taken into account when interpreting the results.

### 4.7 Selection of statistical parameters

In order to compare the results as best as possible, both between CBCT and MDCT as well as in native and contrast enhanced scan, we decided to perform a descriptive evaluation of the exact scores from the different recordings and also to calculate Spearman’s rank correlation coefficient (r_s_) and percent agreement (PA) (Table 2).

PA refers to the proportion of times that two sets of data agree, so in this case how often two observers give the same score for an anatomical structure in one setting and is therefore calculated based on the raw data values for a direct comparison. For the PA a value close to 1 defines a good agreement between the different observers. The advantages of the PA are that it shows an exact match and can be calculated for all structures, even if there is no variance in the data due to a complete match. One disadvantage that cannot be ignored, however, is that the possibility that some agreements could occur purely by chance cannot be taken into account. For this reason we also calculated the r_s_, which portrays the strength and direction of association between two variables from the different devices. A correlation coefficient of $r_{s}$ > 0.4 represents acceptable agreement and $r_{s}$ > 0.7 represents good agreement. In this case for example it indicates if the direction of scoring is the same, even if two observers scored an anatomical structure with different scores. This parameter is calculated based on the ranks of the data rather than the raw data values. Advantages are that r_s_ is robust to outliers and non-linear relationships, but one important disadvantage is that r_s_ cannot be calculated if there is not enough variance in the present data. This is an important fact in our study, as for some structures we were unable to calculate r_s_ due to complete agreement and therefore lack of variance of the data. However, a complete agreement is still a valuable statement for us, as it allows us to say that certain anatomical structures can be represented equally well or poorly in both modalities. That’s why we chose these two parameters, as they each have their advantages and compensate for or take into account the disadvantages of the other parameter in the best possible way.

### 4.8 Limitations

Image acquisition was carried out on fresh cadaveric specimens within 24 h of death, which is why the examinations are comparable to those conducted in recumbent horses under general anesthesia rather than in standing sedated horses. Some disadvantages such as motion artefacts, which are named as major sources for repetition of examinations (13, 24), are therefore excluded from critical assessment. Furthermore, the effect of weight bearing and tension in the tissues on different anatomical structures such as outpouchings and the contribution of contrast medium could not be considered in this study. When comparing these results to findings under clinical conditions, the different properties of injection media must be considered as well. Local anesthetics for example are described to have a lower molecular weight than the here-used contrast medium, which may have influence on the distribution pattern in the tissue (37).

## 5 Conclusion

CBCT imaging represents a valid alternative to conventional MDCT imaging for the three-dimensional assessment of osseous structures of the equine carpal region. Regarding the evaluation of osseous tissues, both modalities yield practically equivalent diagnostic information, while avoiding limitations caused by superimposition. Therefore, and particularly when taking potential advantages regarding the practicability and technical features of CBCT imaging into account, it represents a cost-effective and practical option for diagnostic imaging for issues relating to osseous structures of the equine carpal joint. In cases that require visualization of cartilage, contrast enhancement is unavoidable, and the visualization of these structures is better in MDCT than in CBCT imaging. Soft tissue structure visualization is rather poor, regardless of modality tested here, except for the intraarticular ligaments, which are well visualized after contrast enhancement. As there is too much uncertainty, CBCT can not be recommended for visualization of cartilage and soft tissues in the equine carpal region. On the basis of the present study, further investigations are intended regarding the visualization of pathologies of the carpal region using standing CBCT in patients.

## Data Availability

The original contributions presented in the study are included in the article/supplementary material, further inquiries can be directed to the corresponding author.
